# Effects of Human Neural Stem Cells Overexpressing Neuroligin and Neurexin in a Spinal Cord Injury Model

**DOI:** 10.3390/ijms25168744

**Published:** 2024-08-10

**Authors:** Jiwon Jeong, Yunseo Choi, Narae Kim, Haneul Lee, Eun-Jung Yoon, Dongsun Park

**Affiliations:** 1 Department of Biology Education, Korea National University of Education, Cheongju 28173, Chungbuk, Republic of Korea; yru0805@gmail.com (J.J.); narrr22@gmail.com (N.K.); 2sky1109@gmail.com (H.L.); 2 Department of Life Sports Educator, Kongju National University, Kongju 32588, Chungnam, Republic of Korea; ejyoon@kongju.ac.kr

**Keywords:** synaptic regeneration, synaptic markers, NRXN, NLGN, spinal cord injury, stem cells

## Abstract

Recent studies have highlighted the therapeutic potential of stem cells for various diseases. However, unlike other tissues, brain tissue has a specific structure, consisting of synapses. These synapses not only transmit but also process and refine information. Therefore, synaptic regeneration plays a key role in therapy of neurodegenerative disorders. Neurexins (NRXNs) and neuroligins (NLGNs) are synaptic cell adhesion molecules that connect pre- and postsynaptic neurons at synapses, mediate trans-synaptic signaling, and shape neural network properties by specifying synaptic functions. In this study, we investigated the synaptic regeneration effect of human neural stem cells (NSCs) overexpressing NRXNs (F3.NRXN) and NLGNs (F3.NLGN) in a spinal cord injury model. Overexpression of NRXNs and NLGNs in the neural stem cells upregulated the expression of synaptophysin, PSD95, VAMP2, and synapsin, which are synaptic markers. The BMS scores indicated that the transplantation of F3.NRXN and F3.NLGN enhanced the recovery of locomotor function in adult rodents following spinal cord injury. Transplanted F3.NRXN and F3.NLGN differentiated into neurons and formed a synapse with the host cells in the spinal cord injury mouse model. In addition, F3.NRXN and F3.NLGN cells restored growth factors (GFs) and neurotrophic factors (NFs) and induced the proliferation of host cells. This study suggested that NSCs overexpressing NRXNs and NLGNs could be candidates for cell therapy in spinal cord injuries by facilitating synaptic regeneration.

## 1. Introduction

Spinal cord injury (SCI) induces transient or permanent motor, sensory, or autonomic function interruption. The number of patients with SCI is reported to be more than 250,000 in the United States, with more than 11,000 new cases added annually [1]. SCI research has greatly expanded in recent years, but the mechanisms for functional recovery are not well-understood [2]. However, many researchers have tried to find effective therapies for SCI [3], including stem cell therapy [4,5].

Since stem cells were first identified and characterized, they have been used in therapy for various diseases such as SCI [4,5], traumatic brain injury [6], and neurodegenerative diseases [7]. Since they can self-renew and differentiate into any cell type [8], stem cells and neural progenitor cells are good candidates for SCI [9,10]. Although neurons in the adult central nervous system (CNS) have a low regenerative capacity due to a lack of growth signals [11], after the transplantation of stem cells, the neurons are activated by the upregulation of growth and neurotrophic factors derived from stem cells [12]. However, stem cell transplantation is still not effective in aiding recovery from SCI.

Unlike other tissues, the nervous system comprises specific structures called synapses [13]. SCI, in addition to causing a loss of neuronal cells, alters neuronal connectivity, leading to axonal and synaptic losses [14]. Since synapses transfer and process neural information [13], synaptic regeneration is one of the main goals of SCI therapy [15]. Synapses are connected by neurexins (NRXNs) at the presynaptic neurons and neuroligins (NLGNs) at the postsynaptic neurons [16]. NRXNs and NLGNs are synaptic cell adhesion molecules that form synapses in the pre- and postsynaptic neurons, mediate synaptic signaling, and shape neural network properties by specifying functions [17,18]. Indeed, the synaptic plasticity of these neurons is not related to cognitive functions but rather motor functions [16,19], and decreases after SCI [2]. Therefore, we hypothesized that transplantation of stem cells overexpressing NRXNs or NLGNs into the damaged site would promote SCI recovery by upregulating growth/neurotrophic factors and promoting synapse regeneration.

In this study, we established human neural stem cells (hNSCs) overexpressing NRXNs (F3.NRXN) or NLGNs (F3.NLGN). We then analyzed the expression of other synaptic proteins such as synaptophysin, PSD95, VAMP2, and synapsin in cell and animal models. We investigated the effects of F3.NRXN and F3.NLGN in an SCI mouse model induced by complete transection.

## 2. Results

### 2.1. Construction of F3.NLGN and F3.NRXN Cells

**Figure 1:**
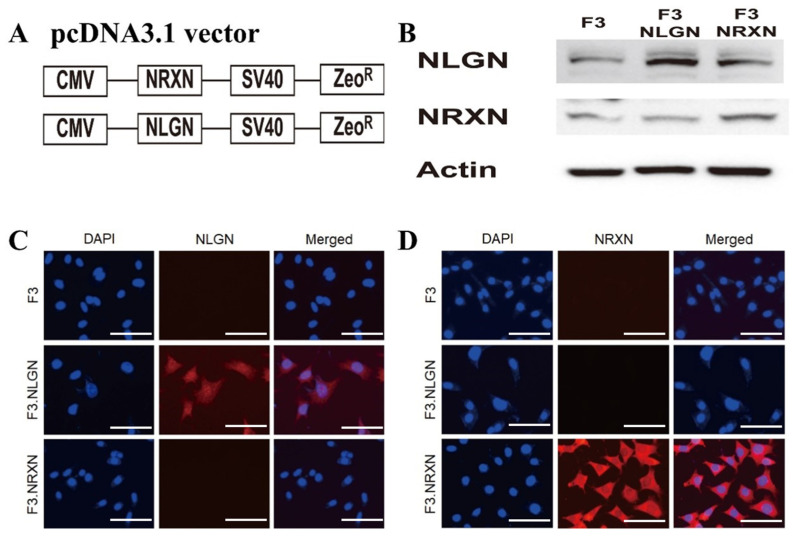
Construction of F3.NLGN and F3.NRXN cells. (**A**) F3.NLGN and F3.NRXN cells were constructed via the transfection of F3 human neural stem cells with a plasmid vector encoding either human NLGNs or NRXNs. (**B**–**D**) Expression of NLGNs and NRXNs in F3.NLGN and F3.NRXN cells assessed via (**B**) Western blotting and (**C**,**D**) immunohistochemistry. Scale bar = 30 μm

The construction of the pcDNA3.1_NLGN and NRXN vectors is shown in Figure 1A. Western blotting analysis confirmed the overexpression of NLGN and NRXN proteins in F3 human NSCs (Figure 1B). Immunocytochemistry also demonstrated strong expression of NLGN and NRXN proteins in F3.NLGN and F3.NRXN cells, respectively (Figure 1C,D).

### 2.2. Expression of Synaptic Markers in F3.NLGN and F3.NRXN Cells

**Figure 2:**
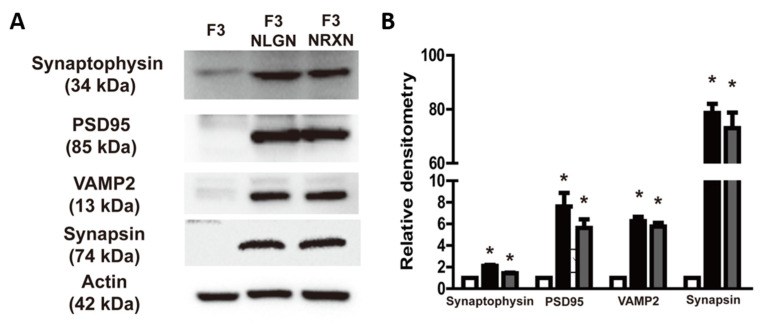
Expression of synaptic markers (synaptophysin, PSD95, VAMP2, and synapsin) in F3.NLGN and F3.NRXN cells. (**A**) Expression of synaptic markers assessed by means of Western blotting. (**B**) Band densities normalized to actin. Densitometric analysis of the Western blot was performed using ImageJ 1.54g. *n* = 3 per treatment group. * Significantly different from F3 cells (*p* < 0.05).

The expression of synaptic markers such as synaptophysin, PSD95, VAMP2, and synapsin was analyzed to confirm whether they were affected by the overexpression of NLGNs and NRXNs. As shown in Figure 2, the expression of these markers was enhanced in F3.NLGN and F3.NRXN cells compared with F3 cells. Interestingly, there was no significant difference between F3.NLGN and F3.NRXN cells.

### 2.3. Effects of F3.NLGN and F3.NRXN Cell Transplantation on Locomotor Function in SCI Mice

**Figure 3:**
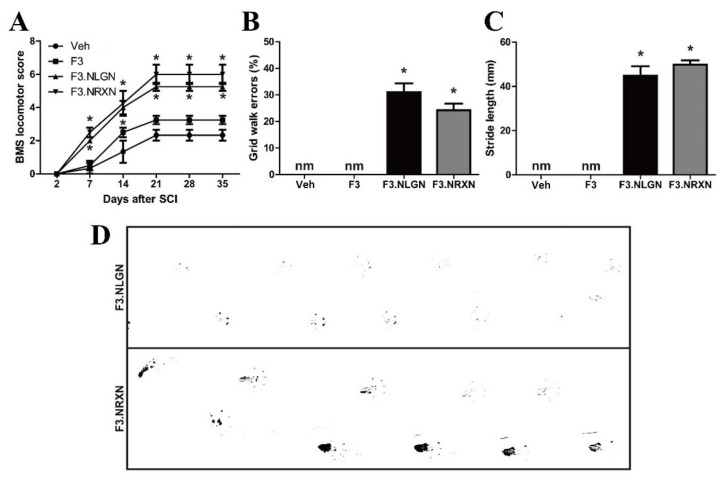
Improvement in locomotor recovery after F3.NLGN and F3.NRXN transplantation in SCI mice. (**A**) Locomotor BMS scores following T7 complete transection SCI. (**B**) Grid walk errors in mice 5 weeks after SCI. (**C**) Stride length of the hindlimbs at 5 weeks after injury in F3.NLGN and F3.NRXN groups. This value could not be measured in the vehicle and F3 groups because they crawled. (**D**) Representative footprints of the hindlimbs in the F3.NLGN and F3.NRXN groups. Data are expressed as mean ± standard deviation. *n* = 10. * Significantly different from the vehicle control (*p* < 0.05). nm, not measured; BMS, Basso Motor Scale; SCI, spinal cord injury.

We evaluated functional recovery in SCI mice by measuring the standardized BMS locomotor scores, grid walk, and footprints of the hind paw. Two days after complete transection, mice showed no observable or slight extensive hindlimb movement (BMS: 0–1), and there were no differences among the four groups (Figure 3A). The recovery reached a stable level approximately 3 weeks after lesioning, and mice generally had some coordination and hind paw rotation when making initial contact with the testing surface and on lift-off. Interestingly, the F3.NLGN and F3.NRXN groups showed a significant increase in BMS scores compared to the vehicle group. However, in mice transplanted with F3 cells, the BMS score increased due to stem cell transplantation compared to the vehicle group, but not significantly. We also performed a grid-walking test by evaluating the incidence of the hindlimbs slipping below the grid plane at 5 weeks after SCI. The F3.NLGN and F3.NRXN groups could walk after stem cell transplantation on the grid, and the percentage of missteps was less than 40% (Figure 3B). However, the vehicle and F3 groups could not walk properly and dragged their hind legs, making it impossible to measure the grid walk error. On the same day, the stride length was measured by inking the feet of mice (Figure 3C,D), recording the gait, and measuring the distance between the hindlimb footprints.

Similarly, the stride length could not be measured in the vehicle and F3 groups, and was ≥40 mm in the F3.NLGN and F3.NRXN groups, showing a significant difference.

### 2.4. Effects of F3.NLGN and F3.NRXN Cell Transplantation on Synaptic Regeneration in SCI Mice

**Figure 4:**
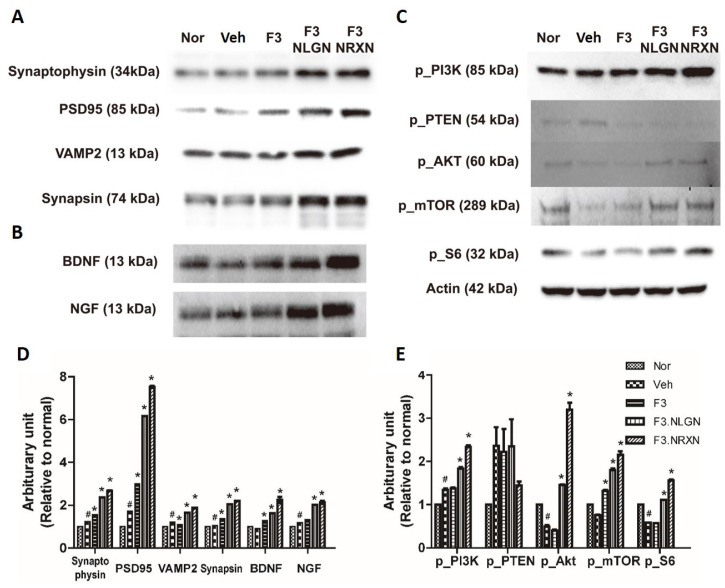
Protein expression after F3.NLGN and F3.NRXN transplantation at the lesion site (2 mm rostral and caudal to injury center) in SCI mice. (**A**) Expression of synaptic markers. (**B**) Expression of BDNF and NGF. (**C**) Expression of the PI3K/PTEN/mTOR signaling pathway after F3.NLGN and F3.NRXN transplantation. Protein expression is analyzed by means of Western blotting. (**D**,**E**) Band densities normalized to actin. Densitometric analysis of the Western blot was performed using ImageJ. *n* = 10 per treatment group. # Significantly different from the normal control (*p* < 0.05). * Significantly different from the vehicle control (*p* < 0.05). SCI, spinal cord injury; BDNF, brain-derived neurotrophic factor; NGF, nerve growth factor.

Since the F3.NLGN and F3.NRXN groups had improved locomotor function compared to the F3 and vehicle groups, we assumed that transplantation of F3.NLGN and F3.NRXN cells increased synaptic regeneration and cell proliferation after SCI. We tested this hypothesis by measuring spinal cord synaptic markers and PTEN-related signaling proteins. As shown in Figure 4A, all synapse regeneration markers significantly increased in the F3.NLGN and F3.NRXN groups compared to the vehicle group. In addition, the transplantation of F3.NLGN and F3.NRXN cells significantly increased the expression of BDNF and NGF. Likewise, transplantation of F3.NLGN and F3.NRXN enhanced the levels of p_PI3K, p_AKT, p_mTOR, and p_S6 in the spinal cord, but reduced that of p_PTEN.

### 2.5. Effects of F3.NLGN and F3.NRXN Cell Transplantation on VAMP2 and Neurofilament Expression in SCI Mice

**Figure 5:**
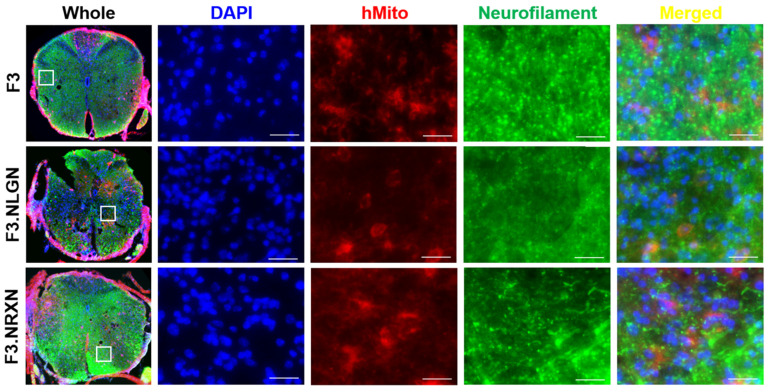
Differentiation of transplanted F3.NLGN and F3.NRXN cells into neurons in SCI mice. hMito (red color) was used as a stem cell marker. Neurofilament (green color) was used as a neuronal marker. DAPI (blue color) was used as a counterstain for the nucleus. Scale bar = 50 μm. SCI, spinal cord injury.

**Figure 6:**
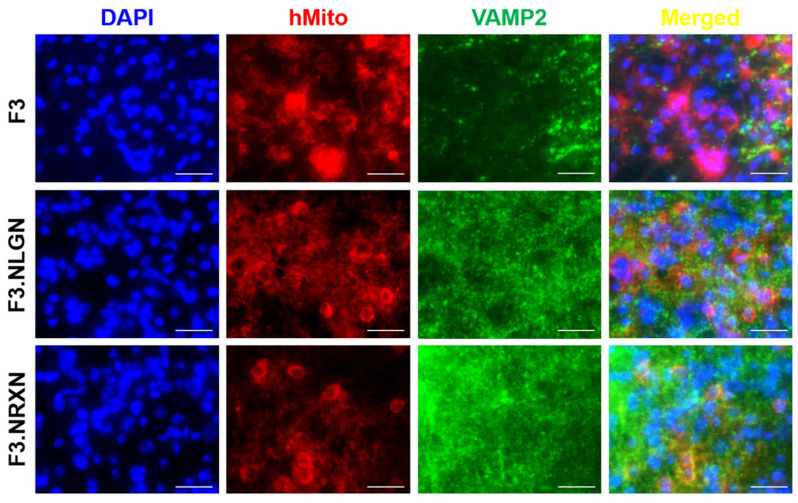
Expression of VAMP2 in transplanted F3.NLGN and F3.NRXN cells in SCI mice. hMito (red color) was used as a stem cell marker. VAMP2 (green color) was used as a synaptic marker. DAPI (blue color) was used as a counterstain for the nucleus. Scale bar = 50 μm. SCI, spinal cord injury.

After double immunostaining, F3.NLGN and F3.NRXN cells were checked at the injury site. As shown in Figure 5, transplanted F3, F3.NLGN, and F3.NRXN cells were observed in the injury site and had differentiated into neurons according to immunostaining results for neurofilament. F3.NLGN and F3.NRXN cells were positive for VAMP2 (Figure 6). Interestingly, F3 cells were also observed in the injury site but were negative for VAMP2.

## 3. Discussion

The utility of stem cells in the treatment and recovery after an SCI has been widely researched [4,5]. Stem cells are attracting attention as prospective treatment options for some disorders, including SCI. Stem cells can replace damaged cells and can differentiate on their own [8]. Additionally, various growth factors produced by stem cells accelerate tissue regeneration [12,20]. Since neurons have a minimal capacity for regeneration and secrete few neurotrophic substances, it is difficult to repair injured neurons [21,22]. Therefore, the major goals of therapeutic techniques, including techniques involving stem cells, are to replace damaged cells and supply growth factors that assist in recovery. In animal models of Alzheimer’s disease, stroke, and SCI, we have previously shown that the transplantation of hNSCs expressing a variety of functional genes involving growth factors helps to maintain host neurons and recover function [7,23,24].

SCI causes direct damage to axons and neuronal cell bodies [22,25]. As a consequence of the injured neuron’s death, synaptic connections are destroyed, resulting in functional loss below the point of injury [26,27]. Restoration of function by means of synapse reconstruction is expected to be a viable therapeutic approach [28]. In this study, we examined the treatment and recovery of SCI, focusing on synapse reconstruction by transplanting stem cells overexpressing NRXN and NLGN proteins involved in connecting neurons at the synapse. NLGNs and NRXNs are essential for synaptic structure and function in the synaptic cleft [16]. Indeed, their expression is increased during synaptogenesis in the cerebellum [29]. NLGNs expressed in non-neuronal cells trigger presynaptic development in contacting axons [30], and regulating their expression influences synaptic markers such as VAMP2, PSD95, and synaptophysin [31,32]. Similarly, this study confirmed that overexpression of NLGNs or NRXNs increased synaptophysin, PSD95, VAMP2, and synapsin levels in NSCs.

In addition, it is well known that SCI causes synaptic changes in the neuronal circuitry within the spinal cord at higher levels over the weeks and months following injury [2,22,33]. As shown in the results, levels of synaptic markers were decreased after SCI, and the mice lost locomotor function. Transplantation of F3 cells increased the BMS score; however, normal gait was impossible in these mice, and they resorted to dragging their hind legs. In comparison, transplantation of F3.NLGN and F3.NRXN cells increased in the BMS, and the gait of these animals improved compared with the SCI animal model. Regarding the immunohistochemistry for neurofilament and VAMP2, the transplanted F3 cells were positive for neurofilament but not VAMP2; in contrast, F3.NLGN and F3.NRXN cells were positive for both neurofilament and VAMP2. After the transplanted NSCs were replaced and differentiated into neurons [34], NSCs overexpressing proteins related to synaptic formation formed synapses with host neurons. Stem cells may be able to differentiate and replace damaged cells after transplantation. However, if they do not form a circuit with host cells, their effect may be insignificant; in contrast, if they form a circuit with host cells like F3.NLGN or F3.NRXN, their function may be significantly restored. In addition to forming circuits with host cells, neurotrophic factors, including NGF and BDNF, are known to play an important role in promoting functional recovery after SCI through either neural protection or neuron regeneration [35,36]. Previous studies have reported that the spontaneous provision of neurotrophic factors through stem cell transplantation can improve recovery from SCI [37]. In this study, NSCs were transplanted in mice to help them recover from SCI. This led to an increase in the expression of neurotrophic factors compared to the vehicle group, resulting in more activation of signaling pathways for cell proliferation. The transplanted F3.NLGN or F3.NRXN cells secreted more BDNF and NGF than F3 cells, and the signaling pathway was activated significantly compared to these cells. Many signaling pathways, including the PI3K/AKT/mTOR pathway, are involved in SCI [38]. As an important intracellular signaling pathway, the PI3K/AKT/mTOR pathway is a good candidate for molecular SCI therapy [39]. The activation of the PI3K/AKT/mTOR pathway is known to regulate cell proliferation, differentiation, and physiological and pathological conditions [40,41]. Many studies have already been conducted targeting this pathway in the treatment of CNS diseases such as stroke [23,42], and the possibility of treatment using its activation in SCI has been suggested [38,39]. Stem cells release BDNF and NGF when transplanted, activating the PI3K/AKT/mTOR signaling pathway and restoring neuronal function.

The mechanism of synaptic remodeling remains unclear owing to the complexity of the nervous system. Interestingly, the degree of recovery, such as locomotor activity and synapse formation, was better in the F3.NRXN group than in the F3.NLGN group. Depending on the characteristics of signal transmission in one direction [43], when neurons are damaged and degenerated due to injury, they do not receive signals below the site of injury, resulting in functional loss [44,45,46]. Although the potential of synaptogenesis is maintained caudal to the lesion [33,47], it requires adequate presynaptic input. Accordingly, recovery of damage caused by SCI is thought to occur more quickly when proteins involved in synapse formation are expressed in presynaptic neurons.

This study investigated the transplantation of F3.NLGN and F3.NRXN cells in SCI mice, which positively impacted motor function. In these mice, the PI3K/AKT/mTOR signaling pathway was activated by BDNF and NGF secreted from transplanted stem cells. NFs promote the differentiation of transplanted stem cells into neurons at the lesion site. The transplantation of stem cells overexpressing functional genes related to synapse formation increased the rate of motor function recovery in SCI mice by promoting synapse formation. This study suggests that hNSC-based therapy using overexpressed NRXN rather than NLGN can effectively restore motor function after SCI, as the degree of recovery was higher in presynaptic neurons.

## 4. Materials and Methods

### 4.1. Construction of F3, F3.NLGN, and F3.NRXN Cells

For the construction of F3.NLGN and F3.NRXN cells, we used HB1.F3 (F3), which is an immortalized human NSC line previously established from primary cultures of a 15-week gestational human fetal brain via infection with a retroviral vector encoding the v-myc oncogene [23]. The full length of NLGN (GQ489206.1) and NRXN (AB035356.1) cDNA was ligated into multiple cloning sites of the pcDNA3.1 vector (Invitrogen, Carlsbad, CA, USA) to generate F3 overexpressing NLGNs and NRXNs. The recombinant plasmids were transformed into DH5α bacteria (Real Biotech Corporation, Banqiao City, Taiwan) and cultured in Luria–Bertani (LB) broth containing 50 μg/mL ampicillin at 37 °C. After inoculation with a single colony in LB medium, plasmid DNA was extracted with a midiprep kit (Qiagen, Hilden, Germany) according to the manufacturer’s protocol. To achieve stable overexpression of NLGNs and NRXNs, F3 cells were plated in 6-well plates before transfection with recombinant plasmid DNA using Lipofectamine 2000 (Invitrogen). Stably transfected colonies were selected using the zeocin (2.0 μg/mL) resistance method (Invitrogen). Western blotting and immunohistochemistry confirmed NLGN and NRXN protein overexpression in each cell.

### 4.2. Cell Culture

F3, F3.NLGN, and F3.NRXN cells were cultured in Dulbecco’s modified Eagle’s medium (DMEM; Biowest, Nuaillé, Cholet, France) containing antibiotics (100 IU/mL penicillin and 100 μg/mL streptomycin) and 10% heat-inactivated fetal bovine serum (Biowest) at 37 °C in a 5% CO_2_/95% air atmosphere. In all experiments, cells were grown until more than 90% confluence was achieved and were subjected to no more than 20 passages.

### 4.3. Animal Model and NSC Transplantation

Eight-week-old male C57BL mice (n = 10/group) were purchased from Daehan Biolink (Eumseong, Republic of Korea). Mice were housed in an environmentally controlled room with a constant temperature (23 ± 3 °C) and relative humidity (50% ± 10%) and a 12 h light/dark cycle and were fed standard rodent chow and purified water ad libitum. After anesthetizing mice with ketamine and rumpun, we approached them dorsally and damaged the T7 area completely using microscissors. After surgery, ampicillin (0.1 mg/kg) and tramadol (0.3 mL/kg) in phosphate-buffered saline (PBS) were injected subcutaneously twice daily in the morning and evening, and urination was performed. Two days after the SCI, F3, F3.NLGN, and F3.NRXN cells were dissolved in an appropriate volume of saline (1 × 10^6^ cells/mouse), intrathecal injection was administered in each mouse. All experimental procedures were approved and carried out according to the Institutional Animal Care and Use Committee of the Korea National University of Education (#KNUE-201908-002-04).

### 4.4. Behavioral Test

The Basso Mouse Scale (BMS) score was measured to evaluate hindlimb motor function [48]. BMS measurements were performed 2, 7, 14, 21, 28, and 35 d after surgery. The BMS scores were evaluated while mice walked in an open field and re-evaluated from digital video documents. Grid walk performance and footprint analyses were carried out 35 d after SCI. The grid walk errors were counted from videotapes played slowly (4 separate trials per test) and averaged across different trials. The stride length on each side and stride width between the two sides of the prints were measured and calculated using multiple steps. For footprint analysis, walking patterns of mouse hind paws were recorded with ink during continuous locomotion across a 50 cm runway.

### 4.5. Western Blot Analysis

Western blotting was performed to analyze protein expression after F3.NLGN and F3.NRXN transplantation. Thirty-five days after SCI, mice were perfused through the heart with cold PBS, and fresh spinal cord tissue containing the injury site (2 mm rostral to and 2 mm caudal to the injury site) was obtained. Collected tissue samples were homogenized in radioimmunoprecipitation cell lysis buffer (Thermo Fisher Scientific, Waltham, MA, USA) with protease inhibitor (Sigma-Aldrich, St. Louis, MO, USA) and phosphatase inhibitors (Sigma-Aldrich). After centrifugation at 15,000 rpm at 4 °C for 15 min, total protein in the supernatant was obtained and quantified using the BCA protein assay kit (Pierce, Rockford, IL, USA). Samples containing the same amount of protein were prepared for Western blots using antibodies against brain-derived neurotrophic factor (BDNF), nerve growth factor (NGF), synaptic markers, proliferation markers, and actin. As synaptic markers, antibodies specific for synaptophysin, PSD95, VAMP2, and synapsin were used. Phosphorylated PI3K (p_PI3K), PTEN (p_PTEN), Akt (p_Akt), mTOR (p_mTOR), and S6 (p_S6) were identified as proliferation markers. The list of antibodies is shown in Supplementary Table S1.

### 4.6. Immunohistochemistry

The mouse spinal cord (n = 2/group), including the NSC transplantation site, was fixed in 4% paraformaldehyde for 24 h, followed by cryoprotection in 30% sucrose–PBS solution for 72 h. The spinal cord was cut into 30 µm slices and immunostained for human mitochondria (hMito; for human cells) as a stem cell marker. VAMP2 was immunostained as a synaptic marker to confirm synaptic formation. For immunohistochemical staining of hMito and VAMP2, spinal cord cryosections were washed in PBS, including 0.01% Tween 20 (PBS-T), and treated with 3% hydrogen peroxide for 10 min to block endoperoxidase activity. After PBS-T washing and blocking with 5% BSA for 30 min, the sections were incubated with primary antibodies overnight at 4 °C, followed by incubation with secondary antibodies conjugated with Alexa Fluor-488 or -594 (1:300, Invitrogen) for 2 h at room temperature. The sections were stained with 4′,6-diamino-2-phenylindole (DAPI, Sigma-Aldrich) to confirm cellular nuclei. All samples were evaluated immediately after staining and photographed using a fluorescence microscope (EVOS FL Auto2 Cell imaging system; Thermo Fisher Scientific).

### 4.7. Statistical Analysis

Statistical comparisons between the groups were performed using one-way analysis of variance (ANOVA) followed by Tukey’s multiple comparison test. All analyses were conducted using SPSS for Windows software (version 12.0; IBM, Armonk, NY, USA). Statistical significance was set at *p* < 0.05. All data are expressed as mean ± standard deviation.

## Data Availability

Data are contained within this article or the Supplementary Materials.

## Supplementary Materials

The following supporting information can be downloaded at https://www.mdpi.com/article/10.3390/ijms25168744/s1.
