# Supplementary material for: Effects of Human Neural Stem Cells Overexpressing Neuroligin and Neurexin in a Spinal Cord Injury Model
Source: Int J Mol Sci. 2024 Aug 10;25(16):8744. doi: 10.3390/ijms25168744 (PMC11354780; doi:10.3390/ijms25168744)
Supplement: Supplementary file 1 [file ijms-25-08744-s001.zip › ijms-3119042-supplementary.pdf]

Supplementary Table S1. List of antibodies used in the current study

Epitope	Company	Cat. Number	Dilution	2° Ab (IgG)
Neuroligin (94 kDa)	Abcam	ab153821	1:1000	Rb
Neurexin (47 kDa)	Bioss	bs-11466R	1:1000	Rb
Synaptophysin (34 kDa)	Abcam	ab32594	1:5000	Rb
PSD95 (95 kDa)	Abcam	ab18258	1:1000	Rb
VAMP2 (13 kDa)	Abcam	ab228996	1:1000	Rb
Synapsin (74 kDa)	Abcam	ab64581	1:1000	Rb
BDNF (13 kDa)	Abcam	ab226843	1:1000	Rb
NGF (13 kDa)	Abcam	ab6199	1:1000	Rb
PI3K (85 kDa)	Cell Signaling Technology	#4292	1:1000	Rb
PTEN (54 kDa)	Cell Signaling Technology	#9554	1:1000	Rb
AKT (60 kDa)	Cell Signaling Technology	#9271	1:1000	Rb
mTOR (289 kDa)	Cell Signaling Technology	#2971	1:1000	Rb
S6 (32 kDa)	Cell Signaling Technology	#2211	1:1000	Rb
Actin (42 kDa)	Cell Signaling Technology	#5125	1:1000	HRP-conjugation

Rb, rabbit; HRP, horseradish peroxidase.
